# Rock falls while high-altitude mountaineering – More often in the last years? Evidence from the Swiss alps

**DOI:** 10.1016/j.heliyon.2024.e25413

**Published:** 2024-01-29

**Authors:** Gasser Benedikt

**Affiliations:** Departement für Sport, Bewegung und Gesundheit, Abteilung Rehabilitative und Regenerative Sportmedizin, Universität Basel, Birsstrasse 320B, CH-4052, Basel, Switzerland

**Keywords:** Permafrost, Melting, Injuries, Emergencies

## Abstract

**Objectives:**

One risk while high-altitude mountaineering despite falls or stranding are rock falls. Due to the climatic change such events might have become potentially more common yielding to the research question to elucidate rock falls while high-altitude mountaineering in the Alps of Switzerland.

**Design:**

A retrospective analysis was conducted from the central registry of the Swiss Alpine Club (SAC) during the observational period from 2009 to 2020.

**Results:**

A total of 266 cases of rock falls was detected during an observational period yielding to in average 22.2 ± 7 emergency cases per year. No increase nor decrease of the number of cases over time (R^2^ = 0.0019) was detected. The average age of a victim was 50.2 ± 18.6 years. The mean of the NACA-Score (NACA = National Advisory Committee for Aeronautics) was 2.7 ± 1.4 implying a moderate injury, however not life threatening. The NACA-Score slightly decreased over time indicating that emergencies have become less severe (R^2^ = 0.1379). The injury pattern was relatively equal distributed between upper and lower extremity.

**Conclusions:**

The slight decrease in the severity of the events might be a consequence of increasing security standards over the observational period. The fact that the average victim of an emergency action was around 50 years, might indicate that stone falls are a constant risk as it can be suggested that alpinists with this age are more risk averse than younger alpinists. As findings were analyzed in a retrospective design, a quasi-prospective design might be helpful while directly analyzing emergencies after occurrence with interviews of involved persons. These hints could be used constructively in order to improve security recommendations.

## 1 Introduction

High-altitude mountaineering has become more and more popular and it is estimated that around 300,000 persons visiting the Swiss Alps per year while high-altitude mountaineering [1,2]. Despite climatic change and other popular risks such as stranding or falls other risks exists in mountain terrain such as rock falls [1,[3], [4], [5]].

Rock falls are not only while recreational activities but in general a considerable risk in the mountains [6]. Thereby, the climatic change influences mass movement activity in mountain environments [[7], [8], [9], [10], [11]]. Rock falls are a the main geomorphological processes influencing the stability of mountain-walls [12]. Rock falls are largely climate-driven, caused by the increasing heat during summer periods predisposing the melting of mountain-wall permafrost [12]. So with the ongoing processes of global warming the degradation of permafrost has to be attributed and the related hazards for people and infrastructure potentially increases [12]. Due to the fact that the Alps are a geologically young mountain area signed by mountain peaks that are quite high and relatively deep valleys, rock falls occur almost everyday [13]. For example, an erosion process started at the end of the Little Ice Age at the west face of the Drus (located in the Mont Blanc massif) was influenced by an erosion dynamic marked by large rock fall events [14]. Analyzes started in the 1950s and showed that the rock failure frequency gradually increased until the large rock fall event (around 300,000 m^3^) in June 2005 [14]. The rock was in the years afterwards specially monitored and a phase of rock failure activity decay was detected until September 2008, a destabilization phase between September 2008 and November 2011, and a new phase of rock failure activity decay from November 2011 to September 2016 [14]. The destabilization phase was thereby signed by three major rock fall events covering a total volume of more than 50,000 m^3 14^. In addition to these three major events of course lots of smaller events were almost weekly occurring [14].

Generally, a constant temperature increase was present in the last decades and many classic routes have changed and peaks are now often absolved on different routes with sometimes more exposed terrain implying that rock falls are a constant risk while being recreationally active [[15], [16], [17]]. To point out, the climatic change yield to the physical weathering of the rock in the Alps and falling rocks are an almost everyday occurrence [8]. In the past, infrastructure was often organized in such a way that known rock fall areas could be avoided^,^ [5,8]. As a consequence of the increase in temperature in the mountains, many areas are increasingly affected by physical weathering through thawing and freezing, which did not take place in the past as simply temperature were mostly below zero [8]. In addition, the decrease in permafrost and retreating glaciers in the Alps expose additional areas which, due to the lack of a stabilizing effect of the ice, can also become potential sources for the formation of rock falls [8].

As hints trying to be developed might help to guide further recommendations concerning security standards of routes analyzing rock fall events in detail might be fruitful allowing to tax risks in the mountains in an adequate manner. This aggravated as climatic change yielded to massive alterations of environmental conditions in the last years. However, unfortunately there is little data about rock fall activities while being recreationally active in the mountains and studies analyzing the impact of rock falls on emergencies in the Swiss alps are missed. The before mentioned directly aims to this study to analyze stone and rockfalls in Switzerland's Alps in the last years. Therefore, we suggested, that possibly the number of stone- and rockfalls did not change over time [18].

## 2 Material & methods

### 2.1 Subjects & tool

High-altitude mountaineering emergency cases from the SAC central registry from 2009 to 2021 were elucidated. The central registry contains data from the Air Glaciers Lauterbrunnen, Air Glaciers Sanenland, the Kantonale Walliser Rettungsorganisation (KWRO), the Snow and Avalanche Research Institute Davos, and the cantonal police registries and the Swiss Air Rescue Service (REGA). Thereby the term *mountain emergency* encompasses all rescue events where mountaineers require professional help of mountain rescue services [15,16]. This also applies to events such as illnesses and evacuations of mountaineers that are not injured. Each mountain emergency includes the emergency number used, date, rescue organization, event, place, canton, activity, NACA (National Advisory Committee for Aeronautics Score) score (Table 1), nationality, birth date, sex, place of residence, coordinates, and a case report [19,20]. Study procedure is performed while using the guidelines respectively regulatory requirements of the local ethics commission and a confirmation concerning anonymous secondary data analysis was received from the local ethics commission (Ethics commission North-eastern Switzerland: 2019-00517).

**Table 1:** NACA-score (National Advisory Committee for Aeronautics score) [19,20].

NACA 0	No injury or disease.
NACA I	Minor disturbance. No medical intervention is required (e.g., slight abrasion).
NACA II	Slight to moderate disturbance. Outpatient medical investigation but usually no emergency medical measures necessary (e.g., fracture of a finger bone, moderate cuts, dehydration).
NACA III	Moderate to severe but not life-threatening disorder. Stationary treatment required, often emergency medical measures on the site (e.g., femur fracture, milder stroke, smoke inhalation).
NACA IV	Serious incident where rapid development into a life-threatening condition cannot be excluded. In the majority of cases, emergency medical care is required (e.g., vertebral injury with neurological deficit, severe asthma attack, drug poisoning).
NACA V	Acute danger (e.g., third grade skull or brain trauma or severe heart attack).
NACA VI	Respiratory and or cardiac arrest.
NACA VII	Death.

### 2.2 Procedure

First, causes of mountain emergencies were classified into categories: falls, being stuck (unable to go further or back), illness, lightning, crevasse accidents, avalanches, stone falls, ice falls (serac), being lost, material failure, other. A rock fall is thereby understood as the sudden fall of rock material [13]. The classification was unique and in consequence multiple classifications were not possible. Missing entries were afterwards analyzed. As missing data in a quantity <5 % are normally not affecting the validity of statements (e.g. < 5 % are missed for age), mean - value imputation was performed [21,22].

### 2.3 Statistical analysis

Mean and Standard deviation for each year for the parameters of age and NACA scores for rock falls were calculated. As the hypothesis of normal distributions for age and NACA scores could not be rejected with Kolmogorov-Smirnov Tests for all subsamples, two-sided heteroscedastic t-tests were performed to detect intersex differences [23]. To detect potential changes over time, linear regressions with calculation of the degree of determination (R^2^) were performed. Calculations were made with Microsoft Excel (Microsoft Inc., Redmond, WA, USA) and SPSS (Armonk, New York, USA).

## 3 Results

**Fig. 1:**
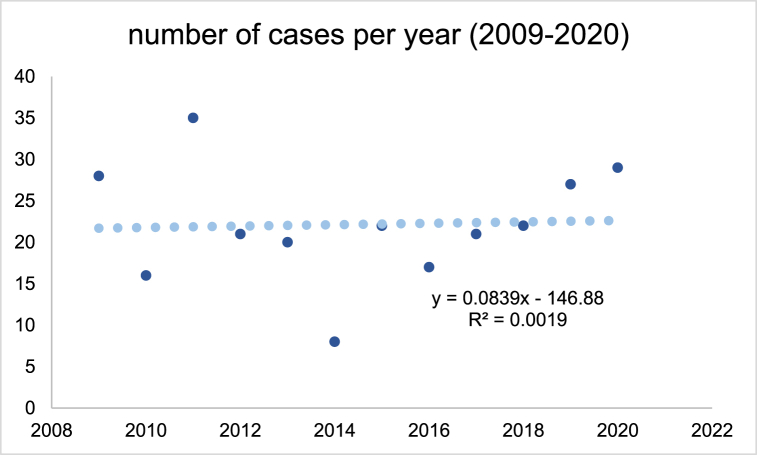
Number of cases over the observational period.

266 cases of rock falls could be detected, whereby 210 male (78.9 %) and 56 (21.1 %) female were identified (Table 2). No significant difference could be detected between male and female concerning NACA-Score and age. Furthermore, as indicated by Fig. 1 which shows the occurrence of cases over the period no change of the number of cases over time was detected (R^2^ = 0.0019).

**Table 2:** Characteristics of the emergency cases identified.

	number of cases	percent	average age (years)	average NACA-Score
**female**	56	21.10 %	50.8 ± 18.6	2.5 ± 1.4
**male**	210	78.90 %	47.8 ± 18.7	2.7 ± 1.6
**total sample**	266	100 %	50.2 ± 18.6	2.7 ± 1.4
**average cases per year**	22.2 ± 7.01		p = 0.285	p = 0.445

**Fig. 2:**
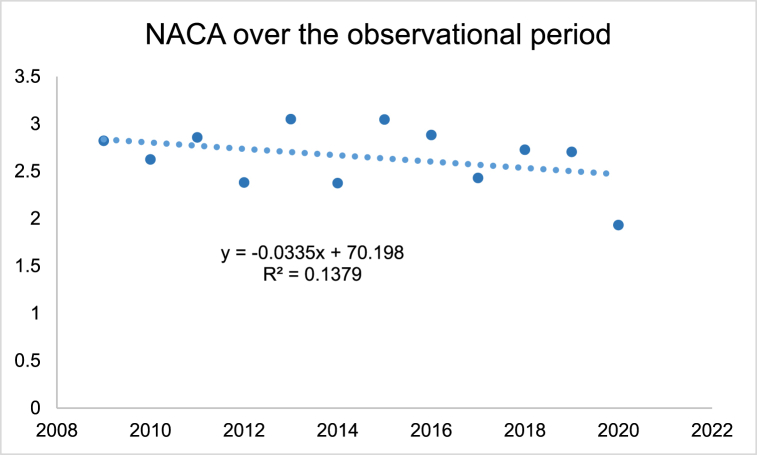
NACA Score over the observational period.

Fig. 2 shows the average NACA Score per year, whereby a slight decrease of NACA Score over time was detectable indicating that emergency events became less severe over time. Over the whole observational period 16 cases had a NACA Score of 7 and were in consequence fatal. Two were female and 14 were men. Average age was 53.1 ± 14.4 years.

**Fig. 3:**
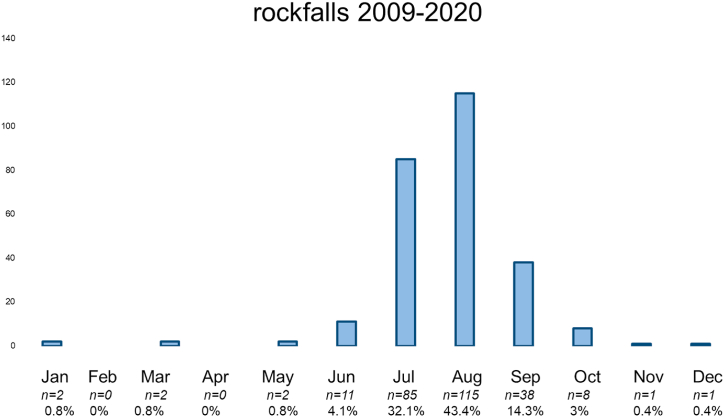
Shows the occurrence over time.

Fig. 3 shows the occurrence per month. Most cases are in the July, August, September (Fig. 3).

**Fig. 4:**
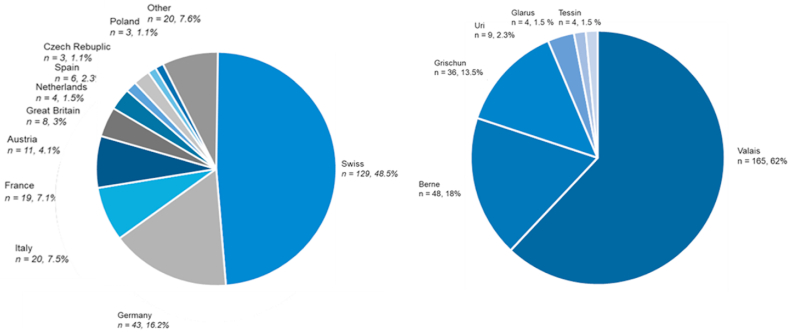
Details Concerning nationality of the victims (a) and Cantone of the events (b).

When trying to capture the peaks 21 cases were on the Matterhorn, 9 cases on the Piz Bernina. In the Mischabel & Allalin region 19 cases, in the Monte Rosa 11 cases. Zinalrothorn and Obergabelhorn count for 18 cases, 8 cases on the Weisshorn, Fletschhorn and Lagginhorn 11 cases, Grand Combin/Dent Blanche/Grand Cornier 12 cases. The sum of the cases on these famous mountains counts for 51.8 % of all cases. The rest was on less known mountains, not necessarily 4000ers. Details concerning the nationality of the victims are shown in Fig. 4a and b.

Concerning the injury pattern Table 3 shows the anatomic location. It gets obvious, that the injuries are almost even distributed over the body, respectively injuries in the lower (40 %) and upper extremity (44.2 %) are equal likely. The Head was in 15 % cases injured.

**Table 3:** Number of cases per anatomical location (multiple mentioning possible).

	anatomic localisation	number of cases	percent
	head	18	15.0
**upper exremity**	shoulder	10	8.3
**44.20 %**	hand	26	21.7
	arm	17	14.2
**lower extremity**	foot	10	8.3
	knee	9	7.5
**40.00 %**	leg	29	24.2
	hip	1	0.8
	**Total**	**120**	**1.0**

## 4 Discussion

It was aimed in this study to analyze emergencies while high-altitude mountaineering due to rock falls in the Alps of Switzerland. Therefore, the mountain emergency services registry from the Swiss Alpine Club was analyzed encompassing all events due to rock falls. The number of cases seems constant during the analyzed period (R^2^ = 0.0019), especially when assuming that while this time the number of alpinists increased [24]. In consequence, when taking the members of the Swiss Alpine Club as proxy (around 4 % more members per year) even a decrease of cases might be suggested [24].

This could be due to preventive activities and to the increased standard of routes with more often drilled bolts on not to rock fall exposed places and in addition higher standards in personal equipment. Furthermore, despite a tendency of a constant melting process of permafrost with for example a peak in the years 2017 and 2018 no association with the number of rock fall emergencies was detected. In consequence, on average around 22 emergency cases could be detected each year with around one to two fatal cases per year making such emergencies a seldom event [25]. Thereby, the total number of 166 cases seems a valid number when comparing with findings from the Austrian alps, which reported for the 13-year timeframe from 2005 to 2018 a number of 229 cases of emergencies due to rock falls [26]. Concerning the severity of an event it is to mention that the average NACA-Score was 2.7 ± 1.43 indicating a moderate to severe injury, however in average not a life-threatening event. Thereby, it has to be considered that potentially a lot of alpinists were able to make the right movement away from the terrain, where potentially the stone would have hit them. Furthermore, alpinists not injured are not captured, respectively alpinists nor alarming the emergency services. The slight tendency of a decrease in the severity of the events might be due to the fact that as already mentioned security standard have improved over the observational period yielding to in consequence less severe events. The fact that the average mountaineer was around 50 years, which are potentially more risk averse than younger alpinists might indicate that stone falls are a constant risk while high-altitude mountaineering and alpinists are during a tour always at risk for such an event. The finding, that most cases were during the months July and August can be explained by the fact that most tours are best absolved in summer [17]. Furthermore, the detection that more than 50 % were on famous mountains (e.g. around 10 % were on the Matterhorn) might imply, that alpinists itself are the cause of the rock falls. Especially on famous routes such as the Hörnligrat on Matterhorn which is well-known for being crowded, a substantial risk comes from early starters potentially harming slower alpinists below them (Fig. 4). Concerning the anatomic location of an injury it is to mention that these not only concerned the head with around 15 % but were relatively even distributed over the upper and lower extremity. In consequence, there seems to be somewhat at random which part of the body is affected in line with the statement, that rock fall events occur with the same likelihood over the observational period exposing the alpinist to a small but constant risk. Concerning the limitations of the study it is to mention that we do not know anything about rock falls that did not yield to an emergency action by the professional emergency services. Furthermore, as the events date sometimes more than ten years back details of the emergencies are difficult to known. A prospective design meaning that directly after an event detail are elucidated might be fruitful in order to deepen the understanding of the mechanisms and the risks that results from rock falls. Preventive recommendations could result and therefore future research might be senseful guided in this direction aiming for a quasi-prospective design while directly elucidating the circumstances of an event. Thereby, once again it must be considered that many events occur on famous mountains such as Matterhorn and in consequence the factor human has to be considered as crucial. As rock falls are often on such famous mountains caused by other mountaineers above, choosing are more seldom route, attacking the peak when it is not to crowded, leaving the hut early when conditions are still frozen and a rockfall event is less likely are all factors that can be affected by the mountaineer's self to increase security. Furthermore, the constant use of for example the REGA-App (allows to locate the mountaineers in a short time and fly exposed persons out of a zone of danger) is recommended as individual preventive measure. This should be accompanied by careful tour planning while for example identifying points of return helping to manage risky moments yielding to adequate choices.

## 5 Conclusiones

The typical victim of a rockfall is a 50-year-old male and is moderately injured. The location of the injury has an equal probability for the upper and lower extremity and in around 15 percent of cases the head is injured. The origin is most likely from a country of the alps and the event is most likely in the Cantone of Wallis where the highest mountains in Switzerland are located. The detected slight decrease in the severity of the events might be a consequence of increasing security standards and the fact that the average victim has an age in the middle of the lifespan might indicate that stone falls are a constant risk as it can be suggested that alpinists around 50 are more risk averse than younger alpinists. As findings were analyzed in a retrospective design, a quasi-prospective design might be helpful while directly analyzing emergencies after occurrence with interviews of victims and other persons being involved that directly observed the rock fall.

## Data availability statement

Despite anonymous data analysis, data is not available. The authors do not have permission to share data. No data associated with the study has been deposited into a publicly available repository.

## CRediT authorship contribution statement

**Gasser Benedikt:** Investigation, Funding acquisition, Formal analysis, Data curation, Conceptualization.

## Declaration of competing interest

The authors declare the following financial interests/personal relationships which may be considered as potential competing interests:Benedikt Andreas Gasser reports was provided by University of Basel Department of Sport Exercise and Health. Benedikt Andreas Gasser reports a relationship with University of Basel that includes: employment. Benedikt Gasser has patent n/a pending to n/a. n/a If there are other authors, they declare that they have no known competing financial interests or personal relationships that could have appeared to influence the work reported in this paper.
