# Supplementary material for: Rock falls while high-altitude mountaineering – More often in the last years? Evidence from the Swiss alps
Source: Heliyon. 2024 Jan 29;10(3):e25413. doi: 10.1016/j.heliyon.2024.e25413 (PMC10847909; doi:10.1016/j.heliyon.2024.e25413)
Supplement: Multimedia component 1 [file mmc1.docx]

**Translation**

Dear Doctor. Gasser

According to Article 3 of the Human Research Act, your research project does not require approval because it is neither research into diseases nor research into the structure and function of the human body. However, if you require a written statement from the EKNZ (e.g. for publication in certain specialist journals), I would ask you to submit a “Jurisdictional inquiry” in BASEC under “Clarification of responsibility”. The study with the number BASEC 2019-00517 will then be deleted again. Kind regards

Veronika Just Veronika Just Albrecht, MSc. | Scientific Secretariat | Ethics Commission Northwestern and Central Switzerland (EKNZ) Hebelstrasse 53 | 4056 Basel | Tel. +41 (0)61 2681353 | Fax. +41 (0)61 2681351 Please note that this email conversation will be attached to your submission in BASEC and will also be archived in the BASEC system.

https://www.fedlex.admin.ch/eli/cc/2013/617/de

Art. 3 Terms

For the purposes of this law:

a.

Research: method-driven search for generalizable findings;

b.

Research on diseases: research into the causes, prevention, diagnosis, therapy and epidemiology of physical and psychological impairments of human health;

c.

Research on the structure and function of the human body: basic research, in particular on the anatomy, physiology and genetics of the human body, as well as non-disease-related research on interventions and effects on the human body;

d.

Research project with expected direct benefit: a research project whose results are expected to improve the health of the participants;

e.

biological material: body substances that come from living people;

f.

health-related personal data: information about an identified or identifiable person relating to their health or illness, including their genetic data;

g.6

genetic data: information obtained through genetic testing about characteristics inherited or acquired during the embryonic phase;

H.

encrypted biological material and encrypted personal health data: biological material and data linked to a specific person via a key;

i.

anonymized biological material and anonymized health-related data: biological material and health-related data that cannot be traced back to a specific person or only with disproportionate effort;

j.

Child: minor up to the age of 14;

k.

Adolescent or young person: minor person from the age of 14;

**Original**

Sehr geehrter Herr Dr. Gasser

Ihr Forschungsprojekt ist nach dem Humanforschungsgesetz Art. 3 nicht bewilligungspflichtig, denn es handelt sich weder um Forschung zu Krankheiten noch um Forschung zum Aufbau und Funktion des menschlichen Körpers. Falls Sie aber eine schriftliche Stellungnahme der EKNZ benötigen (z.B. für eine Publikation in gewissen Fachzeitschriften), bitte ich Sie, in BASEC unter "Clarification of responsibility" eine "Jurisdictional inquiry" einzureichen. Die Studie mit der Nummer BASEC 2019-00517 wird dann wieder gelöscht werden. Freundliche Grüsse Veronika Just

Veronika Just Albrecht, MSc. | Wissenschaftliches Sekretariat |Ethikkommission Nordwest- und Zentralschweiz (EKNZ) Hebelstrasse 53 | 4056 Basel | Tel. +41 (0)61 2681353 | Fax. +41 (0)61 2681351 Bitte nehmen Sie zur Kenntnis, dass diese E-Mail Konversation Ihrer Einreichung im BASEC angefügt und im BASEC System auch archiviert wird.

https://www.fedlex.admin.ch/eli/cc/2013/617/de

Art. 3 Begriffe

Im Sinne dieses Gesetzes gelten als:

a.

Forschung: methodengeleitete Suche nach verallgemeinerbaren Erkenntnissen;

b.

Forschung zu Krankheiten: Forschung über Ursachen, Prävention, Diagnose, Therapie und Epidemiologie von physischen und psychischen Beeinträchtigungen der Gesundheit des Menschen;

c.

Forschung zu Aufbau und Funktion des menschlichen Körpers: Grundlagenforschung, insbesondere zur Anatomie, Physiologie und Genetik des menschlichen Körpers, sowie nicht auf Krankheiten bezogene Forschung zu Eingriffen und Einwirkungen auf den menschlichen Körper;

d.

Forschungsprojekt mit erwartetem direktem Nutzen: ein Forschungsprojekt, dessen Ergebnisse eine Verbesserung der Gesundheit der teilnehmenden Personen erwarten lassen;

e.

biologisches Material: Körpersubstanzen, die von lebenden Personen stammen;

f.

gesundheitsbezogene Personendaten: Informationen über eine bestimmte oder bestimmbare Person, die sich auf deren Gesundheit oder Krankheit beziehen, einschliesslich ihrer genetischen Daten;

g.6

genetische Daten: durch eine genetische Untersuchung gewonnene Informationen über ererbte oder während der Embryonalphase erworbene Eigenschaften;

h.

verschlüsseltes biologisches Material und verschlüsselte gesundheitsbezogene Personendaten: biologisches Material und Daten, die mit einer bestimmten Person über einen Schlüssel verknüpft sind;

i.

anonymisiertes biologisches Material und anonymisierte gesundheitsbezogene Daten: biologisches Material und gesundheitsbezogene Daten, die nicht oder nur mit unverhältnismässigem Aufwand auf eine bestimmte Person zurückgeführt werden können;

j.

Kind: minderjährige Person bis zur Vollendung des 14. Altersjahres;

k.

Jugendliche oder Jugendlicher: minderjährige Person ab der Vollendung des 14. Altersjahres;
